# Lecture

**Published:** 1872-10

**Authors:** 


### SELETIONS.

### LECTURE.

### On Idiosyncrasies.

*Translated from the Lecons de Pathologie Experimentale of M. Claude Bernard, for the Medical and Surgical Reporter.*

Gentlemen:—In my preceding lectures I have endeavored to exhibit the intimate relations existing between physiology and pathology, and to prove, as far as it was possible for me to do, that the phenomena which are manifested in the living body, either in a healthy or in a diseased condition, should in all cases receive a rational interpretation. I have told you that they may be directly referred to the action of biological laws which govern the vital functions, not only in the normal state, but also in all the perturbations which these functions may experience.

Daily experience, nevertheless, teaches us that morbid causes, may be, otherwise, their general effects, are far from acting with equal intensity on the various individuals exposed to their influence. Cold, hunger, thirst, fatigue and moral distress are the most ordinary causes of disease. Are they not, to a certain degree, the lot of human nature? How then does it happen that among those exposed to their daily action some sink so rapidly, whilst others resist with great energy? And when an epidemic rages at a given spot, how is it tha the prevailing disease attacks only certain persons, sparing other individuals who are in constant communication with the sick? To this apparently mysterious power, which thus modifies in each particular case the influence of external agents, we give the name of *Idiosyncrasy*.

We may, I think, admit as a principle that predispositions, not only morbid, but likewise physiological, exist in men as well as in animals. In the normal state each individual, by virtue of his peculiar organization, is more especially exposed than any other to certain peculiar accidents. The various animals subjected to our experiments are far from exhibiting the same phenomena under the influence of the same agents. You already know that as we rise or descend in the animal scale, we meet with animals more or less sensible to the action of certain poisons; of those, for example, which act more particularly on the nervous system. Thus it will be seen that even in the limits of health, living beings may present striking differences. As we have already demonstrated, these different properties do not depend solely on the general organization of the animal, but frequently also on the conditions in which it has been placed. In this way a rabbit may be reduced to the physiological level of a batrachian; and by reversing the experiment, we arrive at an opposite result. Now these important modifications almost always bear a close relation to the state of the nervous system.

It is not alone among animals belonging to different species that we remark great inequalities in this respect. It frequently happens that individuals belonging to the *same species* resemble each other so little that it is impossible to subject them to the same experiments. The nervous sensibility of a hunting dog is so highly developed that the slightest operation gives rise in it to fever which may be followed by a fatal termination. These animals are therefore unsuitable for experiments on the gestric juice, the pancreatic secretion, or other similar phenomena; every operation performed on the interior of the abdominal cavity may excite peritonitis in these eminently sensitive animals. How vast the difference between them and the dogs which may be considered as belonging to an inferior breed. The results of the experiments are totally various. During the operation the animal scarcely makes a movement, apparently does not suffer at all; the appetite is unchanged, the secretions remain natural. In short, the various functions of the economy follow their accustomed course despite the pain inflicted.

In the horse these differences are, if possible, still more pronounced. The characteristics of certain breeds are, in common parlance, attributed to *blood*; it would be more exact to attribute them to the *nerves;* in fact, it is a highly irritable, sensitive, nervous organization which distinguished a blooded beast from one of the half savage little horses which inhabit mountainous regions. The results of the same ex per iment would be diametrically opposite in those animals, and no comparison could be established between them. Therefore whenever an experiment requires great powers of resistance in the subject, the latter must be selected from the inferior races. If nervous irritability and exquisite sensibility are, on the contrary, the necessary qualities, we must have recourse to the more highly bred animal. Experiments on recurrent sensibility which succeed almost always in the hunting dog, fail almost constantly when performed on the shepherd dog. In this respect cold-blooded animals are found in the lowest degree of the scale. It will thus be conceived that a state which, in certain individuals, constitutes an actual disease, may, in others, harmonize with the natural conditions of life.

We may be allowed to believe that in man, the differences which separate individuals, ought to be still greater than those between the various species of animals; and if we may be permitted to allude to questions which, at this moment, are occupying public attention, is it not true that hynotism is a peculiar state which can be developed only in a small number of impressionable and nervous individuals? Are not the phenomena of somnambulism to be classed in the same category? It is therefore evident that idiosyncrasies are merely peculiar sensibilities which exist in a normal condition in various individuals.

Hitherto we have considered merely physiological, and so to speak, innate predispositions; however, as physicians, the accidental, morbid and transitory idiosyncrasies are those which most interest us. To ascertain the conditions from which they may arise is, for the physiologist, a study of the last degree of importance.

If we compare an animal in a state of abstinence with another in that of active digestion, the most evident dissimilarities will be remarked in the results of all the experiments to which they may be subjected. A dose of strychnia which kills the latter immediately, will require a certain space of time to act on the former. The absorbent power has naturally been quoted to explain so striking a discrepancy; but do we not know that in the fasting state absorption is infinitely more active than during the process of digestion?

This explanation can not therefore be sustained. The debilitation of the physical properties of the nervous system is in reality the only cause to which in this case we can appeal Deprived of nutrition, the animal gradually descends in the scale, and at last acquires properties more or less removed from its original condition. Is that an actual disease? Undoubtedly not, it is the natural result of a well-known physiological condition.

We are, therefore, authorized to deny absolutely the existance of a so-called *morbid physiology*, if by these words is understood a state of things completely independent of the ordinary laws of life. Expressions used in a limited sense should be banished from scientific language; they serve only to confuse one's ideas and to mislead observers. When we speak of medical chemistry, for example, we do not pretend to say that the chemical reactions which supervene in the vital phenomena are subjected to laws differing from those which exist outside of the living being. Morbid physiolygy has undoubtedly processes which may be peculiar to it, but its laws are perfectly identical with those which govern, in the healthy state, the functions of life.

Not inanition alone thus modifies the conditions of life; cold, and many other causes likewise act in the same way, and vary the results of our vivisections. At a low temperature cold blooded animals become less and less sensible to the action of certain poisons; a stronger dose of strychnia is required to kill a frog in winter than in summer. But chloroform, ether, and even the ordinary intoxication of alcohol, produce similar effects; and it is generally believed in America that intoxication is a preservive against the bite of the rattlesnake.

Such are the physiological modifications of the economy which lead us to the study of those which belong more especially to the pathological condition. It has for a long time been known that remedies do not act on the sick in the same manner as on the individual in high health. Now, the biological conditions which determine the disease are evidently the source of these irregularities. To give a well-known example, we observe that wine, brandy, and the various alcoholic preparations so liberally used by American physicians in the treatment of fevers, when administered in doses which in a healthy man would inevitably produce intoxication, appear to cause an entirely different effect on the patient. The fact may be explained in two modes: in the first place, the absorbent function might be, during the disease, modified, or even suspended; in the second place, the nervous system might be powerfully depressed. We know, for instance, that in certain cases of typhoid fever, absorption remains benumbed for a considerable length of time. This is proved by administering to the patient small doses of prussiate of potassa, of which no traces can be found in the urine, nor in the other secretions. An analogous condition may be artificially produced; for when the secretions are over-excited, the surfaces lose a portion of their properties. The internal surface of the salivary glands, which absorb so rapidly strychnia or curare in a state of repose, become momentarily rebellious to the action of these poisons during the process of secretion. We have seen a dog die almost instantly after the injection of five cubic centimetres of an aqueous solution of strychnia into the duct of Wharton. On a repetition of the same experiment on an animal in which the salivary secretion was excited by galvanism, death supervened only after an interval of twelve minutes.

Chloroform furnishes us a pathological example of the same fact; so long as this abundant serous excretion is maintained, the intestinal parietes can absorb no remedy. It will be said that these modifications are determined by the disease; this is undoubtedly true, but a peculiar physiological process is developed under its influence, and the facts observed flow from it as a natural consequence.

The suppression of the absorbing power is manifested in conditions entirely different from those which we have just been studying; it has been observed in acute mania; the sole cause of it, in this case, appears to reside in the nervous system, for so soon as the crisis has passed, absorption recommences, as in the healthy state.

Curare has been recently used in the treatment of tetanus; of four cases, two were cured and two died. In the favorable cases, the ordinary effects, or, more correctly speaking, the physiological effects of the poison, manifested themselves; nothing similar took place in those who died. They presented, perhaps, a special condition of the nervous system, which prevented the manifestation of the action of this substance. It may be supposed that had they been placed under treatment at a less advanced stage of the disease, the result might have been more happy. In connection with this we may cite the well-known effects of the sulphate of quinine, which, given in a large dose, depresses the pulse, and acts on other morbid phenomena only by modifying the nervous system, and producing a more or less intense degree of deafness, etc. This physiological effect is most intimately related to its therapeutic action, for if the dose be too rapidly diminished the deafness disappears, and the original morbid phenomena regain their violence.

But if we find, in animals as in man, various predispositions which may modify the action of remedies, we likewise find some which expose them to entirely different diseases under the influence of absolutely similar causes. Such is the section of the filaments of the sympathetic nerve. I lately intended to perform some experiments on animals which had been for a long time deprived of food. I therefore allowed dogs which had been used for operations on the sympathetic nerve to starve for several days; but on the occurrence of cold weather the animals died most unexpectedly; the autopsy revealed in one a pneumonia, pleurisy in another, and enteritis in the two remaining. We ascertained that, though placed in identical external conditions, these animals had been attacked by entirely different diseases, corresponding to the regions in which the sympathetic nerve had been wounded. When a rabbit is submitted to absolute inanition, life is ordinarily prolonged from fifteen to twenty days, but when certain filaments of the great sympathetic have been previously divided, these animals, deprived of nourishment, perish in a few days, from acute inflamation of the viscera which are fed by the nervous branches which have been severed. When I began this series of experiments, now some time ago, I perceived that the section of even the larger branches of the great sympathetic seemed to produce no special pathological condition in these animals, so long as their general health remained in fact. I have seen females conceive and bring forth their young in spite of the operation they had undergone, but so soon as the economy was profoundly debilitated by the want of nourishment the viscera deprived of their nerves became the seat of acute inflamations. I had, therefore, succeeded in creating, artificially, peculiar idiosyncrasies in animals, and I could predict with absolute certainty that, the health being once undetermined, a morbid condition would be developed at a certain point.

Pathological predispositions should, therefore, be considered as special physiological conditions, which, in the majority of cases, depend on nervous system, and medicine would have made an immense step if it were possible to foresee, in a state of health, the various morbid predispositions, and thus to predict the approach of danger. A Russian army surgeon, who had invented a new sphygmograph, and had applied it to the study of various diseases, pretended to have ascertained, during an epidemic of cholera, a peculiar feebleness of the pulse, which manifested itself for several days before the invasion of the malady, in those about to be attacked by it. I am not aware if these observations have been corroborated by others, but it would certainly be of immense advantage to be able to point out beforehand, during an epidemic, which individuals are most liable to fall under its influence. It would then be very easy to prescribe preventive measures, and establish hygienic regulations.

To conclude, I think that I have a right to advance the proposition that idiosyncrasies are not mysterious powers residing in the bosom of our organs, nor functions entirely new, superadded, as it were, to those which already existed. In them are to be seen only the simple manifestations of the ordinary laws of physiology.

